# O Pré-Condicionamento com Dexmedetomidina Reduz a Lesão de Isquemia-Reperfusão do Miocárdio em Ratos, Inibindo a Via PERK

**DOI:** 10.36660/abc.20200672

**Published:** 2021-09-24

**Authors:** YuJiao Chen, Song Cao, Hui Chen, CunZhi Yin, XinPeng Xu, ZaiQun Yang

**Affiliations:** 1 Zunyi Medical University Zunyi Guizhou China Zunyi Medical University, Zunyi, Guizhou – China; 2 Affiliated Hospital North Sichuan Medical College NanChong SiChuan China Affiliated Hospital of North Sichuan Medical College, NanChong, SiChuan - China

**Keywords:** Dexmedetomidina, cardiopatia Isquêmica, mortalidade, Reperfusão Miocárdica, Pre-Condicionamento, Hipnoticos e Sedativos, Proteínas Quinases, Ratos

## Abstract

**Fundamento:**

A cardiopatia isquêmica atraiu muito atenção devido às altas taxas de mortalidade, custos do tratamento e a crescente morbidade na população jovem. Estratégias de reperfusão reduziram a mortalidade. Porém, a reperfusão pode levar à morte do cardiomiócito e subsequente dano irreversível ao miocárdio. No momento, não há um tratamento eficiente e direcionado para a lesão de isquemia-reperfusão (I/R).

**Objetivos:**

Avaliar se a dexmedetomidina (DEX) tem efeito protetivo na I/R do miocárdio e explorar os possíveis mecanismos por trás dela.

**Métodos:**

Corações de ratos foram perfundidos com o sistema de perfusão de Langendorff e aleatoriamente distribuídos em cinco grupos: grupo controle, perfundido com solução de Krebs-Henseleit (K-H) por 205 minutos sem isquemia; e quatro grupos de teste que foram submetidos a 40 minutos de isquemia global e 120 minutos de reperfusão. O Grupo DEX, o grupo ioimbina (IO) e o grupo DEX + IO foram perfundidos com DEX (10 nM), IO (1 μM) ou a combinação de DEX e IO antes da reperfusão, respectivamente. A hemodinâmica cardíaca, o tamanho do infarto do miocárdio e a histologia do miocárdio foram avaliados. A expressão da proteína-78 regulada pela glicose (GRP78), a proteína quinase do retículo endoplasmático (PERK), a PERK fosforilada, o fator de iniciação eucariótico 2α (eIF2α), eIF2α fosforilado, o fator de transcrição 4 (TCF-4) e a proteína homóloga à proteína ligadora do acentuador CCAAT (CHOP) foram avaliados. *P<* 0,05 foi considerado para indicar a diferença estatisticamente significativa.

**Resultados:**

O pré-condicionamento com DEX melhorou a função cardíaca nos corações com I/R, reduziu o infarto do miocárdio, a apoptose do miocárdio e a expressão de GRP78, p-PERK, eIF2α, p-eIF2α, TCF-4 e CHOP.

**Conclusões:**

O pré-tratamento com DEX reduziu a lesão de I/R no miocárdio ao suprimir a apoptose, o que foi induzido pela via PERK.

## Introdução

A cardiopatia isquêmica tem atraído muita atenção devido às suas altas taxas de mortalidade, custos do tratamento e a crescente morbidade na população jovem. Nas últimas três décadas, estratégias de reperfusão para aliviar a lesão isquêmica têm sido incluídas na prática clínica. Porém, a reperfusão pode levar à morte do cardiomiócito e subsequente dano irreversível ao miocárdio.^1^

A lesão de isquemia-reperfusão do miocárdio é um processo fisiopatológico de longo prazo e complexo. O processo de restabelecer o fluxo sanguíneo ao miocárdio isquêmico pode levar a uma grande variedade de mudanças biológicas básicas, incluindo deficiência na homeostase iônica, ruptura de radicais de oxigênio, autofagia, disfunções metabólicas do trifosfato do ATP, estresse oxidativo, disfunção mitocondrial e apoptose.^2^ Estudos recentes demonstraram que o dano causado ao miocárdio envolve dois processos: lesão de isquemia-repercussão (I/R). A lesão de I/R é um grande determinante da mortalidade de longo prazo; por isso, a possibilidade de aliviar a extensão da lesão tem grande valor individual e socioeconômico.^3 - 7^

Foi demonstrado que a apoptose do cardiomiócito é um importante mecanismo da lesão de I/R do miocárdio.^8^ A apoptose é acionada pelo receptor mitocondrial e da morte nas condições da reperfusão miocárdica.^9^ Porém, esses mecanismos ainda precisam ser determinados.^2^ Estudos recentes exploraram a associação entre o retículo endoplasmático (RE) e a lesão de I/R do miocárdio.^9 - 11^ Condições de estresse, como I/R, hipóxia e estresse oxidativo foram identificados na desregulação das funções do RE, levando ao estresse do RE.^12^ As consequências patológicas da ruptura do lúmen do RE e da falta de comunicação foram implicadas na lesão de I/R do miocárdio.^13 , 14^ Porém, não está claro se o estresse do RE induz a apoptose ao ativar a PERK durante a lesão de reperfusão do miocárdio.^15^

No momento, devido à falta de medicamentos específicos e terapias padronizadas, não há tratamento preciso e direcionado para a lesão de I/R. A dexmedetomidina (DEX) é agonista do receptor α_2_ adrenérgico (RA), e tem impacto limitado na hemodinâmica e na respiração; por isso, promove a sedação e analgesia ideais para pacientes submetidos à cirurgia cardiovascular.^16^ Estudos anteriores demonstraram que o pré-condicionamento com DEX trouxe efeitos cardioprotetores em corações isquêmicos.^17 - 20^ Porém, o mecanismo molecular da proteção da DEX, até hoje, é desconhecido. Este estudo avaliou a hipótese de que o pré-tratamento com DEX protege o coração da lesão de I/R por meio da ativação da PERK, o que depende da ativação do receptor α_2_. Assim, este estudo usou um modelo de coração isolado com I/R para avaliar os efeitos da DEX na lesão da I/R e seu potencial mecanismo.

## Métodos

### Experimento com animais

Ratos machos e adultos da linhagem Sprague-Dawley (8 a 10 semanas de idade; 260-280g) foram obtidos no centro de experimento com animais de Changsha (Changsha, China). Todos os procedimentos experimentais estavam de acordo com o “Guia para o Cuidado e Uso de Animais de Laboratório”, publicado na China (n. 1492, 2001), e foram aprovados pelo Comitê de Cuidado e Uso de Animais Experimentais da Zunyi Medical University. Todos os ratos foram acomodados em condições padrão (temperatura ambiente, 22ºC, ciclo claro/escuro de 12 horas), com acesso livre à comida e à água, no laboratório Guizhou Key Laboratory of Anesthesiology and Organ Protection, da Zunyi Medical University.

### Isolamento dos corações dos ratos adultos

Os ratos foram anestesiados com pentobarbital sódico (45 mg/kg, via intraperitoneal). Depois do sucesso da anestesia, o coração foi imediatamente retirado do peito por meio da esternotomia e imerso em solução gelada de Krebs-Henseleit (K-H) (NaCl, 119 mM; KCl, 6,0 mM; CaCl_2_, 1,24 mM; NaHCO_3_, 20,1 mM; KH_2_PO_4_, 1,24 mM; MgSO_4_, 1,24 mM; glicose, 11,2 mM). O coração foi perfundido de modo retrógrado usando o método de Langendorff pela aorta (PanLab). A solução de K-H foi perfundida sob constante pressão de perfusão de 75-80 mmHg, e balanceada com uma mistura de 95% de O_2_ e 5% de CO_2_, com pH de 7,35-7,45 a 37°C. Um balão de látex conectado a um transdutor de pressão foi inserido no ventrículo esquerdo por meio da válvula mitral, e preenchido com soro fisiológico para produzir pressão diastólica final do ventrículo esquerdo (PDFVE) de 2-10 mmHg. O volume do balão foi mantido constante durante o experimento. A frequência cardíaca (FR), a PDFVE, a pressão desenvolvida no ventrículo esquerdo (PDVE) (PDVE = pressão sistólica no ventrículo esquerdo (PSVE) – PDFVE), a pressão ventricular esquerda máxima de mudanças positivas e negativas (±dp/dt_max)_ e o produto de taxa de pressão (PTP = FC x PDVE) foram registrados pelo sistema Lab Chart.

### I/R e pré-condicionamento com DEX em corações isolados de ratos

**Figura 1:**
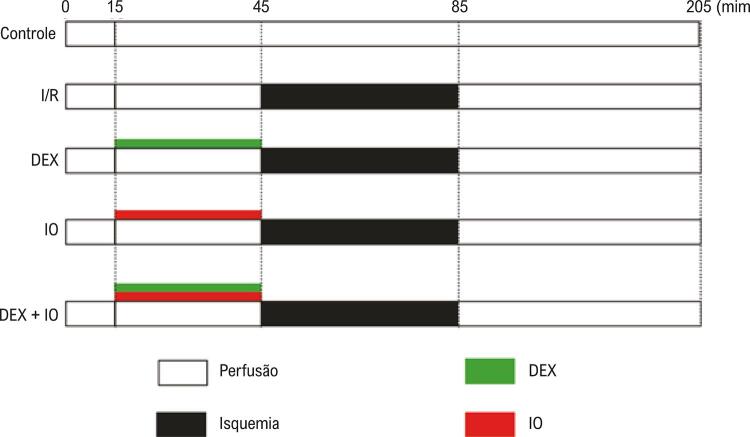
– *Modelo de Langendorff da lesão de isquemia-reperfusão do miocárdio. Para cada teste, após 15 minutos de balanceamento da perfusão, os corações preparados foram aleatoriamente distribuídos em cinco grupos para igualar a PDVE e a FC, que estavam a menos de 50 mmHg e 200 batidas/min. As amostras no grupo controle eram continuamente perfundidas com solução de K-H por 205 minutos. As amostras nos outros quatro grupos foram perfundidas por 30 minutos antes dos 40 minutos da isquemia global normotérmica, seguidos de 120 minutos de reperfusão. As amostras no grupo I/R não tiveram protocolo de pré-condicionamento e foram perfundidas com solução normal de K-H antes da isquemia; as amostras no grupo DEX foram perfundidas com DEX (10 nM) antes da isquemia; as amostras no grupo IO foram perfundidas com IO (1 μM) antes da isquemia; as amostras do grupo DEX + IO foram perfundidas com DEX (10 nM)+IO (1 μM) antes da isquemia. PDVE: pressão desenvolvida no ventrículo esquerdo, FC: frequência cardíaca, K-H: Krebs-Henseleit, I/R: isquemia-reperfusão, DEX: dexmedetomidina, IO: ioimbina*

Para cada teste, após balancear a perfusão por 15 minutos, os corações cuja PDVE de base e FC eram >50mmHg e >200 batidas/min foram aleatoriamente distribuídos em cinco grupos, em envelopes lacrados. As amostras em todos os grupos foram perfundidas por 30 minutos antes dos 40 minutos de isquemia global normotérmica, seguido de 120 minutos de reperfusão, exceto no grupo controle (n=6): (i) grupo controle, sem protocolos de pré-condicionamento, perfundidos com solução de K-H normal; (ii), grupo de I/R, sem protocolos de pré-condicionamento, perfundidos com solução de K-H normal antes da isquemia; (iii) grupo DEX, pré-condicionado com 10 nM de DEX antes da isquemia; (iv) grupo ioimbina (IO), pré-condicionado com 1 μM IO antes da isquemia; e (v) grupo DEX + IO, pré-condicionado com 10 nM DEX + 1 μM IO antes da isquemia. As concentrações de DEX (Hengrui pharmaceutical) e do antagonista foram selecionadas com base em estudos anteriores. A ioimbina (IO) foi usada como antagonista do receptor α_2_ adrenérgico. DEX e IO foram dissolvidas em solução modificada de Krebs-Ringer ( Figura 1 ).

### Determinação do tamanho do infarto

O tamanho do infarto foi avaliado com a coloração do cloreto de trifeniltetrazólio (TTC). Depois da reperfusão, os tecidos que aderiram à raiz arterial foram removidos, e todo o coração foi congelado. O coração congelado foi cortado em fatias de 1-2 mm. As fatias foram imersas em um frasco com 1% de TTC e incubadas em banho-maria por 30 minutos, a 37ºC. O frasco era continuamente agitado para obter a coloração equilibrada. Então, as fatias foram colocadas em formaldeído 10% por 24 horas, transferidas para uma placa de vidro e uma tampa de vidro foi colocada sobre as fatias. Calços de 2 mm foram colocados nas laterais, entre os vidros, para obter a grossura da fatia, e foram tiradas fotografias digitais. As áreas do infarto e as áreas normais foram determinadas de forma cega, por meio da planimetria, com o software Image-Pro Plus 6.0 (Media Cybernetics, Inc.), e o tamanho do infarto foi demonstrado como um percentual da área total.

### Exame histopatológico

Os tecidos do miocárdio coletados dos ratos foram cortados em seções de 1 cm, fixados em paraformaldeído 4% e embebidos em parafina. Para quantificar a extensão do dano ao miocárdio, as biópsias foram recortadas em seções de 5 µm, que foram coradas com hematoxilina e eosina (HE) por 90 minutos, em temperatura ambiente, e seis campos de visão (ampliação, x200) foram selecionados aleatoriamente para avaliação em cada grupo.

### Método TUNEL

O método de marcação de terminações dUTP e deoxinucleotidil terminal transferase (TUNEL) foi realizado para determinar a extensão da apoptose no miocárdio utilizando um kit comercializado (Baiao Cisco Biological Technology), de acordo com as instruções do fabricante. A marcação nuclear verde foi definida para células TUNEL positivas. Para determinar a extensão da apoptose do miocárdio, cinco campos (ampliação, x200) foram selecionados aleatoriamente de duas seções em cada grupo, e o índice apoptótico (IA) foi calculado por meio do software Image-Pro Plus 6.0 (AI): número de células apoptóticas / número total de células contadas.

### Western blot

O coração foi homogeneizado e lisado com uma solução tampão RIPA (Thermo Fisher scientific, Inc). Os tecidos foram centrifugados a 125 x g por seis minutos, e o sobrenadante foi coletado. A concentração da proteína foi determinada pelo método BCA (Thermo Fisher scientific, Inc.). As amostras foram separadas em eletroforese em gel de poliacrilamida-dodecil sulfato de sódio 10% e transferidas para membranas de nitrocelulose. As membranas foram bloqueadas com leite desnatado por duas horas. As membranas foram analisadas com os anticorpos primários anti-GRP78 (1:1000; cat n. PA1-014A; Thermo Fisher scientific), anti-PERK (1:1000; cat n. PA5-79193; Thermo Fisher scientific), anti-p-PERK (1:1000; cat n. MA5-15033; Thermo Fisher scientific), anti-eIF2α (1:1000; cat n. MA1-079; Thermo Fisher scientific), anti-p-eIF2α (1:1000; cat n. 44-728G; Thermo Fisher scientific), anti-TCF-4 (1:1000; cat n. PA5-68802; Thermo Fisher scientific), anti-CHOP (1:1000; cat n. PA5-86145; Thermo Fisher scientific), anti-GAPDH (1:1000; cat n. MA5-32539; Thermo Fisher scientific) a 4ºC durante a noite. Depois de lavar as membranas três vezes, as amostras foram incubadas com anticorpos secundários conjugados com peroxidase de rábano (1:1000; cat n. G-21234; Thermo Fisher scientific) por duas horas. O conteúdo da proteína foi determinado pelo sistema de imagem em gel (Tanon Science & Technology).

### Análise estatística

O tamanho da amostra do estudo foi baseado em um experimento preliminar. As médias de PDFVE no grupo controle, no grupo DEX e no grupo de I/R, seguido da reperfusão por 120 minutos foram 12,52; 16,23; 22,39, respectivamente, e o desvio padrão foi de 1,45; 2,44 e 145, respectivamente. O poder (1 – β) foi considerado como 0,8. Para os grupos controle e DEX, o tamanho da amostra era seis para cada grupo. Para os grupos I/R e DEX, a amostra era composta de quatro em cada grupo. Então, o tamanho de amostra de seis foi determinado por grupo. Os dados são apresentados como média ± desvio padrão (DP). O SPSS 20.0 (IBM Corp) foi utilizado para a análise estatística. As diferenças entre os grupos foram analisadas com a análise de variância (ANOVA). O teste de Shapiro-Wilk foi utilizado para verificar a normalidade. O teste de diferença mínima (LSD) foi usado para comparar a homogeneidade da variância, e o teste T3 de Dunnett foi aplicado para comparar a variância heterogênea. P *<* 0,05 foi considerado para indicar a diferença estatisticamente significante.

## Resultados

### DEX melhora a recuperação cardíaca da lesão de I/R

**Figura 2:**
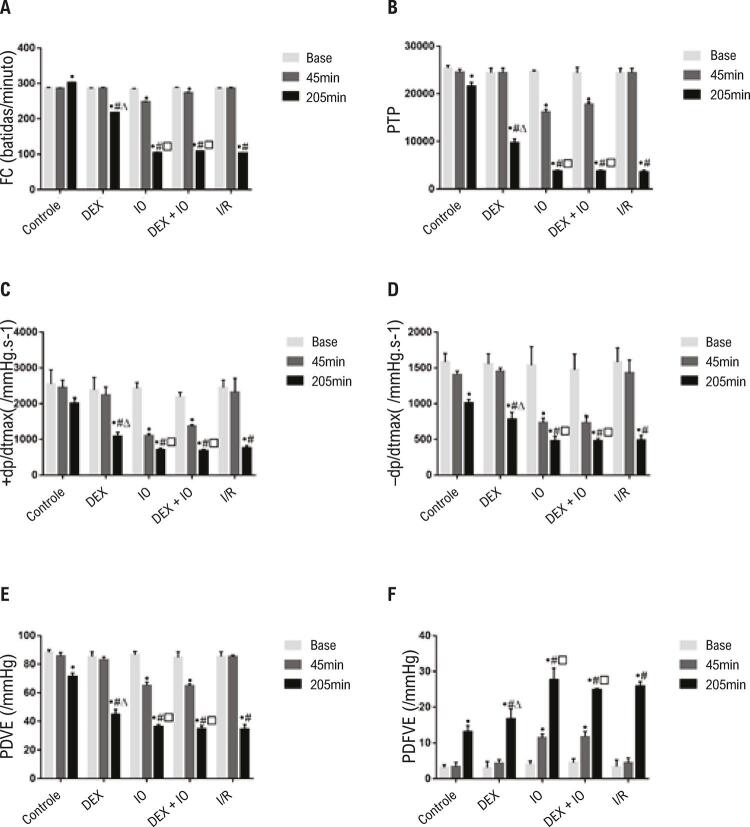
– *A dexmedetomidina melhora a função cardíaca em corações de ratos com isquemia-reperfusão. A-F: efeito da dexmedetomidina na FC, produto de taxa de pressão (PTP = FC + PDVE), pressão ventricular esquerda máxima de mudanças positivas e negativas (±dp/dt_max_) da lesão de I/R em ratos, PDVE, PDFVE. Os dados são apresentados como média ± desvio padrão. n=12. ^•^P<0,05, vs. ponto de início da isquemia, ^#^P<0,05, vs. grupo controle na reperfusão por 120 minutos. ^∆^P<0,05, vs. grupo I/R com reperfusão por 120 minutos. ^^P<0,05, vs. grupo DEX com reperfusão por 120 minutos. Base: no final do ponto de equilíbrio, 45 minutos: ponto de início da isquemia, 205 minutos: 120 minutos de reperfusão. FC: frequência cardíaca; I/R: isquemia-reperfusão; PDVE: pressão desenvolvida no ventrículo esquerdo; PTP: produto de taxa de pressão; ±dp/dt_max:_ pressão ventricular esquerda máxima de mudanças positivas e negativas; PDFVE: pressão diastólica final do ventrículo esquerdo, DEX: dexmedetomidina.*

Para determinar os efeitos da DEX na lesão de I/R do miocárdio, a preparação isolada do coração perfundido foi realizada ao perfundir corações de ratos por 30 minutos, com solução modificada de K-H, antes de 40 minutos de isquemia global, seguidos de 120 minutos de reperfusão. A análise de variância demonstrou que não houve diferenças significativas na hemodinâmica entre os grupos ao final do ponto de equilíbrio. Depois de 45 minutos de reperfusão, não houve diferenças significativas na FC, ±dp/dt_max_, PDVE, PDFVE e PTP, exceto no grupo IO e no grupo DEX + IO. No grupo controle, FC, ±dp/dt_max,_ PDVE e PTP foram maiores, e a PDFVE foi menor em comparação aos outros grupos após a reperfusão por 120 minutos. Isso sugere que a isquemia de 40 minutos em temperatura ambiente foi desafiadora para os corações dos ratos. Durante a segunda metade da reperfusão de 120 minutos, muitos corações exibiram falência e arritmia (~10%). Porém, no grupo DEX, FC, ±dp/dt_max_, PDVE e PTP ficaram maiores, e a PDFVE ficou menor em comparação aos outros grupos após a reperfusão por 120 minutos, exceto o grupo controle. Assim, o pré-condicionamento com DEX melhorou significativamente a hemodinâmica após a lesão de I/R do miocárdio, e os efeitos foram revertidos pelo efeito da ioimbina (IO) como antagonista do receptor adrenérgico ( Figura 2 ).

### Pré-tratamento com DEX reduz o tamanho do infarto do miocárdio

**Figura 3:**
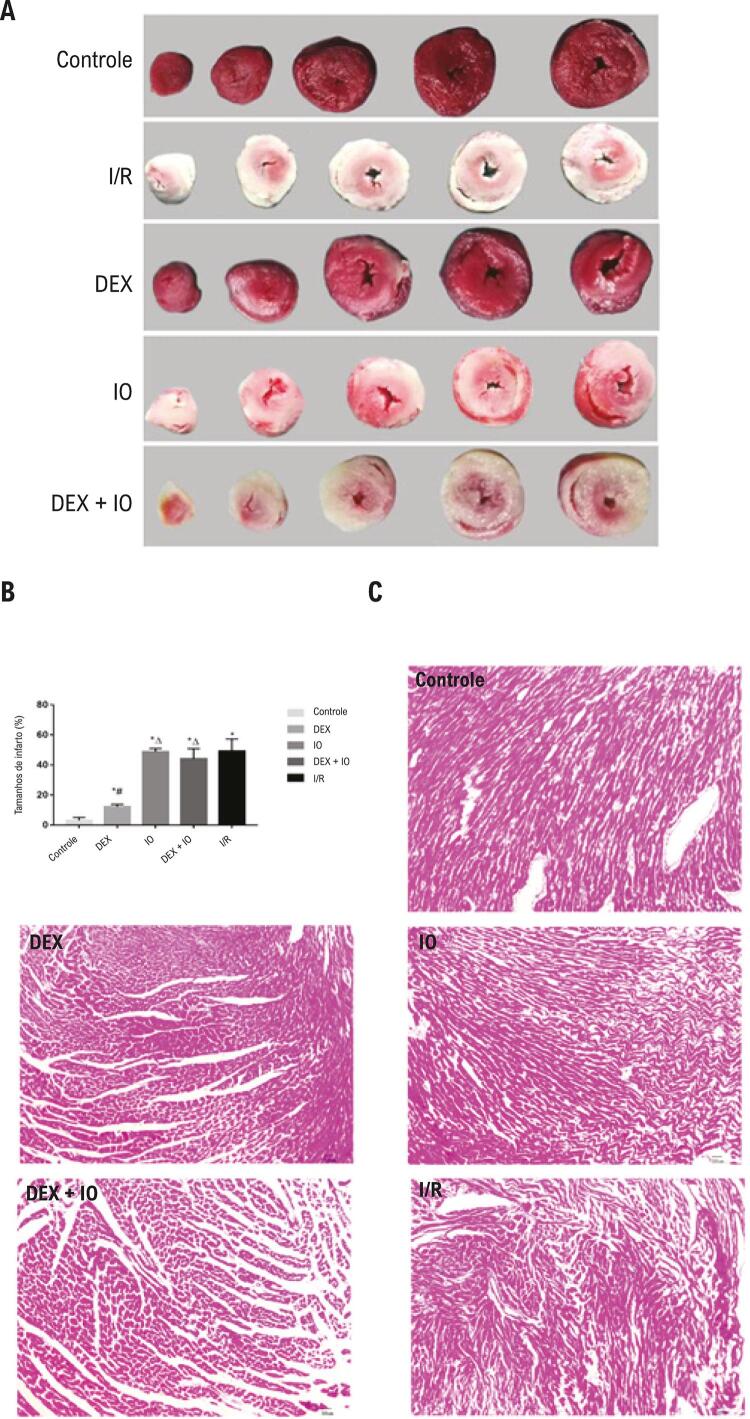
– Coloração com TTC e HE no método Langendorff após a lesão de isquemia-reperfusão. (A) Imagens representando as amostras coloradas com TTC mostrando a área do infarto (branco) e a área sem infarto (vermelho). (B) Análise do tamanho do infarto do miocárdio no controle e no coração isolado com I/R induzida. Os dados são apresentados como média ± desvio padrão. N = 6^. *^P<0,05, vs. grupo controle; ^#^P<0,05, vs. Grupo da I/R; ^∆^P<0,05, vs. grupo DEX. (C) Imagens representando as amostras com coloração de HE (ampliação, ×200) demonstrando mudanças histopatológicas no miocárdio. TTC: coloração do cloreto de trifeniltetrazólio. HE: hematoxilina e eosina, I/R: isquemia-reperfusão.

O método TTC de coloração, que é padrão ouro para testes de infarto do miocárdio, foi usado para avaliar a função cardioprotetora da DEX. No grupo controle, a porcentagem de infarto foi baixa; porém, aumentou significativamente nos grupos I/R, IO e DEX + IO ( Figura 3A ). Em ratos pré-tratados com DEX, a área do infarto foi significativamente menor em comparação ao grupo I/R. Além disso, não houve diferenças entre os grupos IO e DEX + IO em comparação ao grupo I/R. Assim, a adição de IO reverteu os efeitos protetores da DEX na lesão do miocárdio ( Figura 3 ).

### Pré-tratamento com DEX atenua a lesão no tecido do miocárdio

A lesão miocárdica foi medida por meio da coloração com HE. A coloração com HE revelou que a estrutura do miocárdio estava completa e exibia um arranjo regular, fibras musculares cardíacas normais e nenhuma necrose, com um leve edema no cardiomiócito no grupo controle, enquanto a estrutura do miocárdio foi severamente comprometida depois da lesão de I/R. O grupo I/R mostrou arranjo irregular de miofibrilas e músculos cardíacos rompidos. Como demonstrado na Figura 3C , o pré-condicionamento com DEX melhorou muito essas alterações patológicas; porém, a IO reverteu parcialmente o efeito protetor.

### Pré-tratamento com DEX suprime a apoptose miocárdica

**Figura 4:**
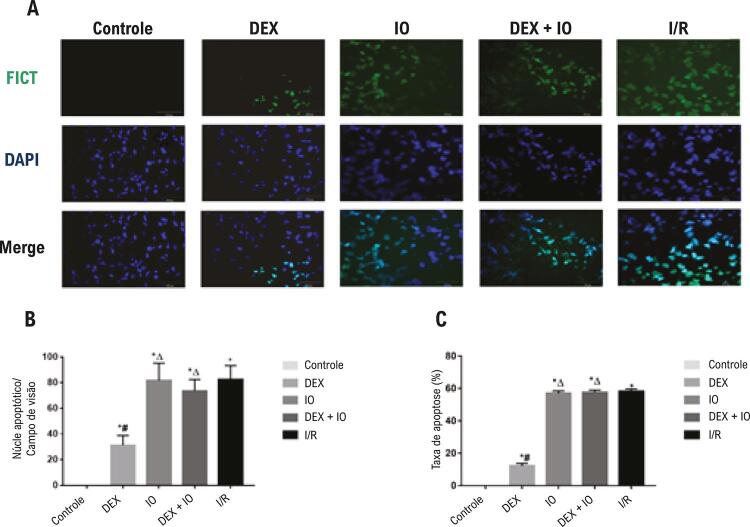
– Análise TUNEL para detectar apoptose em corações com isquemia-reperfusão. (A) Imagens representativas das análises TUNEL (ampliação, x400) mostrando o núcleo total (azul) e o núcleo apoptótico (verde). (B) Análise do núcleo apoptótico do cardiomiócito no grupo controle e no coração isolado com I/R induzida. (C) Análise das taxas de apoptose do cardiomiócito no grupo controle e no coração isolado com I/R induzida. Dados são apresentados como média ± desvio padrão. N=6. ^*^P<0,05, vs. grupo controle; ^#^P<0,05, vs. grupo I/R; ^∆^P<0,05, vs. grupo DEX. TUNEL: método de marcação de terminações dUTP e deoxinucleotidil terminal transferase.

O método de TUNEL foi usado para determinar os efeitos da DEX na apoptose do cardiomiócito em corações isolados de ratos após I/R. Em comparação ao grupo controle (taxa de apoptose, 0,00±0.00%), o número de células TUNEL positivas aumentou significativamente nos grupos de I/R (taxa de apoptose, 58,17±0,60%), IO (taxa de apoptose, 57,11±1,39%) e DEX + IO (taxa de apoptose 57,62±1,50%); ao mesmo tempo, não houve diferenças significativas na taxa de apoptose entre os grupos I/R, IO e DEX + IO ( Figura 4C ). Em comparação aos grupos I/R, IO e DEX + IO, a taxa de apoptose diminuiu muito no grupo DEX. Assim, a DEX pode reduzir a apoptose do miocárdio. Porém, a IO reverteu completamente este efeito ( Figura 4 ).

### O pré-tratamento com DEX alivia a apoptose ao inibir o estresse do RE via PERK

**Figura 5:**
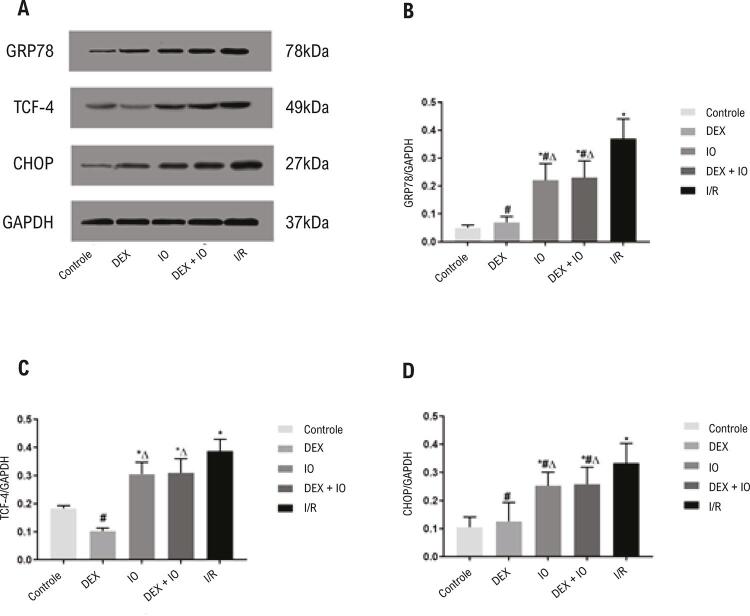
– A dexmedetomidina protegeu o miocárdio da lesão de I/R e reduziu a apoptose. (A) Teste Western blot detectou expressão das proteínas GRP78, TCF-4 e CHOP. (B-D) Análise da expressão de TCF-4, GRP78 e CHOP no controle e no coração isolado com I/R induzida. Os dados são apresentados como média ± desvio padrão. n=6. *P<0,05, vs. grupo controle. #P<0,05, vs. grupo I/R, ∆P<0,05, vs. grupo DEX. I/R: isquemia-reperfusão. GRP78: proteína-78 regulada pela glicose, TCF-4: fator de transcrição 4, CHOP: proteína homóloga à proteína ligadora do acentuador CCAAT.

**Figura 6:**
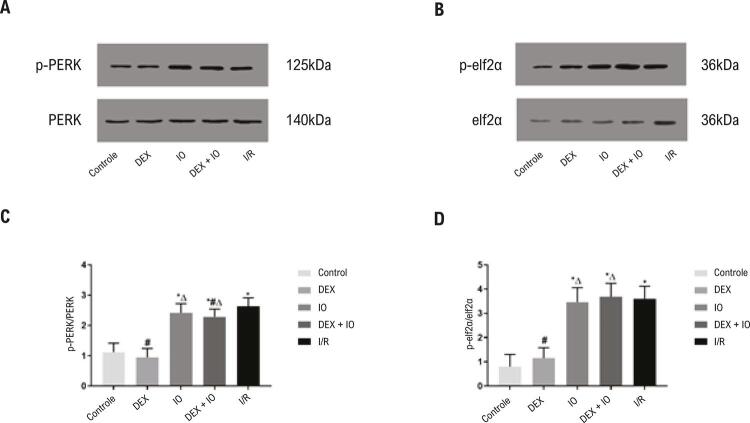
– Efeito da DEX na expressão de p-PERK e p-eIF2α. (A, B) Western-blot detectou a expressão das proteínas PERK, p-PERK, eIF2α, p-eIF2α. (C, D) Análise da expressão de PERK, p-PERK, eIF2α, p-eIF2α no controle e no coração isolado com I/R induzida. Dados são apresentados como ± desvio padrão. n=6. ^*^P<0,05, vs. grupo controle. ^#^P<0,05, vs. grupo I/R, ^∆^P<0,05, vs. grupo DEX. I/R: isquemia-reperfusão. PERK: proteína quinase do retículo endoplasmático, p-PERK: proteína quinase do retículo endoplasmático fosforilada, eIF2a: fator de iniciação eucariótico 2α, p-eIF2α: fator de iniciação eucariótico 2α fosforilado.

Para esclarecer o estabelecimento de sucesso de um modelo de estresse do RE induzido pela lesão de I/R do miocárdio, a ativação da GRP78 e da PERK foi avaliada. Neste estudo, a GRP78, importante marcador da ocorrência do estresse do RE, foi altamente regulada no nível da proteína em todos os grupos experimentais em comparação ao grupo controle ( Figura 5 ). Como a GRP78 é alvo direto da PERK, que é um transdutor altamente conservado da apoptose no estresse do RE, os níveis de expressão das proteínas PERK e p-PERK foram examinados. Sob condições fisiológicas, a superexpressão da GRP78 não alterou a ativação da PERK como sendo indiretamente determinada pela forforilação da PERK ( Figura 6 ). A expressão da proteína p-PERK nos grupos I/R, IO e DEX + IO foi maior se comparada àquela no grupo controle e nos grupos DEX ( Figure 6 ). Em contraste, ela foi bloqueada com sucesso pelo tratamento com DEX. Esses resultados sugerem que a DEX pode ter participação na proteção gerada pelo estresse do RE contra a lesão de reperfusão, enquanto o efeito protetivo da DEX foi revertido pela IO, em comparação ao grupo DEX + IO. Para avaliar melhor os efeitos da DEX na via PERK e seu potencial mecanismo molecular, componentes derivados da PERK na resposta de estresse do RE foram investigados, incluindo o fator de iniciação eucariótico 2α (eIF2α), ativando o fator de transcrição 4 (TCF-4) e proteína homóloga à proteína ligadora do acentuador CCAAT (CHOP). Os resultados demonstraram que os níveis de p-eIF2α, TCF-4 e CHOP foram regulados nos grupos I/R, IO e DEX + IO, em comparação ao grupo controle, enquanto o pré-tratamento com a DEX inibiu a expressão das três proteínas ( Figura 5 , Figura 6 ). A análise do modelo TUNEL confirmou esses resultados. Assim, os resultados mostraram que a DEX pode aliviar a lesão de I/R do miocárdio mediada pela apoptose ao suprimir a ativação da via PERK.

## Discussão

Este estudo indicou que a DEX pode proteger contra a lesão de I/R do miocárdio, o que está de acordo com um estudo anterior.^21^ Os efeitos protetivos da DEX na lesão de I/R do miocárdio por meio da inibição das vias apoptóticas foram confirmados previamente.^22^ Da mesma forma, os resultados deste estudo indicaram que a DEX melhorou a hemodinâmica cardíaca e reduziu a apoptose, o que foi revelado pelas mudanças na morfologia apoptótica e nas taxas de apoptose do método TUNEL. O estresse excessivo do RE demonstrou ter um papel importante na lesão de I/R do miocárdio, levando à apoptose.^23^ GRP78, que é uma proteína acompanhante do RE, pertence à família das proteínas do choque térmico (Hsp70), o que indica a ocorrência do estresse do RE e regula a homeostase do RE em certo ponto.^24^ Estudos anteriores demonstraram que a I/R induz a expressão da proteína GRP78.^25 , 26^ Os resultados deste estudo demonstraram que a proteína GRP78 foi induzida pela I/R, enquanto o tratamento com DEX reduziu a expressão de GRP78, o que indicou que a DEX pode exercer seus efeitos protetores ao suprimir o estresse do RE.

A PERK é um receptor de estresse com atividade de serina-treonina quinase, que é ativada por trans-autofosforilação e oligomerização. Em condições prolongadas de estresse do RE, a via PERK/eIF2α/TCF-4/CHOP contribui com a apoptose durante a lesão de I/R do miocárdio.^27 , 28^ Um estudo prévio reportou que a GRP78 localizada na membrana é essencial para a fosforilação da PERK,^26^ o que também foi observado neste estudo. Observa-se que a maior expressão da proteína GRP78 foi detectada no miocárdio do coração de rato isolado e perfundido com isquemia por 40 minutos e reperfusão por 120 minutos.^29^ Além disso, descobrimos que a isquemia por 40 minutos foi ótima para os corações com isquemia-reperfusão no método Langendorff;^30^ então, selecionamos isquemia por 40 minutos e reperfusão de 120 minutos no modelo atual *ex vivo* da lesão do estresse do retículo endoplasmático (ERE). Também foi reportado que a ativação do domínio PERK quinase nos primeiros estágios do estresse do RE leva à fosforilação da eIF2α, o que reduz a iniciação da transcrição e o enovelamento de proteínas, mantendo a homeostase no retículo endoplasmático. A fosforilação avançada da eIF2α por meio da ativação da TCF-4 induz a superexpressão da proteína apoptótica CHOP, levando à apoptose.^31 , 32^ Estudos relacionados mostraram que os mecanismos da DEX contra a lesão de I/R do miocárdio são mediados pela ativação da α_2_-Ars e dos processos anti-inflamatórios,^22^ enquanto o efeito da DEX no estresse do RE na lesão de I/R do miocárdio foi raramente investigado. Os resultados deste estudo demonstraram que a DEX inibiu o estresse do RE mediado pelas vias PERK/eIF2α/TCF-4/CHOP durante a lesão de I/R do miocárdio. Um estudo anterior descreveu que a I/R do miocárdio causa um acúmulo anormal de proteínas não enoveladas no lúmen do retículo endoplasmático, e contribui para a autofosforilação da PERK.^33^ Neste estudo, o nível de expressão da proteína p-PERK aumentou no grupo I/R, mas diminuiu com o pré-tratamento com a DEX. Com a ativação da PERK, a fosforilação da proteína derivada eIF2α inibiu o agrupamento de ribossomos 80S e a síntese proteica em massa.^34 , 35^ Outro estudo indicou que a desfosforilação da eIF2α suprimiu a sinalização dos derivados da TCF-4/CHOP.^36^ Assim, a eIF2α pode ser um componente chave na sinalização da PERK. Os resultados deste estudo demonstraram que depois do tratamento com DEX, o nível de p-eIF2α diminui em relação ao grupo I/R. A TCF-4 tem papel essencial na promoção da apoptose, que é induzida pela fosforilação da eIF2α.^37 - 39^ Os resultados deste estudo sugerem que a TCF-4 pode ser responsável pela apoptose do cardiomiócito durante o estresse do RE induzido pela I/R, enquanto o tratamento com a DEX pode diminuir a TCF-4. CHOP, que é membro da família de fatores de transcrição da proteína ligadora do acentuador CCAAT,^40^ sendo um marcador clássico da iniciação da apoptose, ou seja, tem papel essencial na apoptose do miocárdio mediada pelo estresse do RE.^41 , 42^ Nossos resultados sugerem que a expressão da proteína CHOP aumentou no grupo I/R, o que corresponde aos resultados da análise da apoptose. Este estudo demonstrou que a DEX tinha um efeito anti-apoptótico, e o mecanismo pode depender do estresse do RE ativado pela via PERK.

A IO é um bloqueador seletivo do α_2_-AR pré-sináptico, que dilata o músculo liso vascular, reduz o tom simpático e aumenta o tom parassimpático periférico. Neste estudo, a IO reverteu os efeitos protetores do pré-tratamento com a DEX no miocárdio de ratos, o que resultou em maior tamanho de infarto do miocárdio e apoptose do cardiomiócito; além disso, os níveis de expressão das proteínas PERK aumentaram. Juntos, esses resultados indicam que o efeito protetor do pré-condicionamento com a DEX em miocárdios de ratos pode ser antagonizado por um bloqueador RA, o que está de acordo com um estudo prévio.^43^

Embora os resultados deste estudo identifiquem um papel importante da via PERK na sobrevivência de cardiomiócitos, houve algumas limitações. Mais trabalhos são necessários para avaliar outros potenciais mecanismos além da apoptose na via PERK. Além disso, a apoptose deve ocorrer no contexto de excessivo estresse do RE. Ao contrário, o RE promove a sobrevivência celular normal ao oferecer nutrientes de organelas e proteínas digestivas danificadas e quando os níveis de estresse do RE estão baixos, chamado autofagia do RE induzida pelo estresse. Outras avaliações são necessárias para determinar o limite entre a autoadaptação protetora da PERK e a apoptose prejudicial na lesão de I/R do miocárdio.

## Conclusão

Este estudo confirmou que o pré-condicionamento com a DEX reduziu a lesão de I/R do miocárdio e melhorou a função cardíaca ao inibir a via apoptótica ativada pela PERK.
