# Supplementary material for: O Pré-Condicionamento com Dexmedetomidina Reduz a Lesão de Isquemia-Reperfusão do Miocárdio em Ratos, Inibindo a Via PERK
Source: Arq Bras Cardiol. 2021 Sep 24;117(6):1134–44. [Article in Portuguese] doi: 10.36660/abc.20200672 (PMC8757152; doi:10.36660/abc.20200672)
Supplement: Translation

# Translation: Dexmedetomidine Preconditioning Reduces Myocardial Ischemia-Reperfusion Injury in Rats by Inhibiting the PERK Pathway

**Authors:** YuJiao Chen, Song Cao, Hui Chen, CunZhi Yin, XinPeng Xu, ZaiQun Yang

**Affiliations:** 1 China Zunyi Medical University, Zunyi, Guizhou – China; 2 China Affiliated Hospital of North Sichuan Medical College, NanChong, SiChuan - China

**Keywords:** Dexmedetomidine, Ischemic Cardiomyopathy/mortality, Myocardial Reperfusion, Hipnotics and Sedatives, Protein Kinases, Rats

## Abstract

**Background:**

Ischemic heart disease has attracted much attention due to its high mortality rates, treatment costs and the increasing morbidity in the young population. Strategies for reperfusion have reduced mortality. However, reperfusion can lead to cardiomyocyte death and subsequent irreversible myocardial damage. At present, the timely and targeted treatment of ischemia-reperfusion (I/R) injury is often lacking.

**Objectives:**

To evaluate if dexmedetomidine (DEX) has a protective effect in myocardiual I/R and explore the possible mechanism behind it.

**Methods:**

Rat hearts were perfused with a Langendorff perfusion system, and randomly assigned to five groups: control group, perfused with Krebs-Henseleit (K-H) solution for 205 minutes without ischemia; and four test groups that underwent 40 minutes of global ischemia and 120 min of reperfusion. The DEX group, the yohimbine (YOH) group and the DEX + YOH group were perfused with DEX (10 nM), YOH (1 μM) or the combination of DEX and YOH prior to reperfusion, respectively. Cardiac hemodynamics, myocardial infarct size, and myocardial histology were evaluated. The expression of glucose-related protein 78 (GRP78), protein kinase R-like ER kinase (PERK), phosphorylated PERK, eukaryotic initiation factor 2α (eIF2α), phosphorylated eIF2α, activating transcription factor 4 (ATF4), and CCAAT/enhancer-binding protein homologous protein (CHOP) were assessed. P<0.05 was considered to indicate a statistically significant difference.

**Results:**

DEX preconditioning improved the cardiac function of I/R hearts, reduced myocardial infarction, myocardial apoptosis, and the expression of GRP78, p-PERK, eIF2α, p-eIF2α, ATF4 and CHOP.

**Conclusions:**

DEX pretreatment reduced myocardial I/R injury by suppressing apoptosis, which was induced by the PERK pathway.

## Introduction

Ischemic heart disease has attracted much attention due to its high mortality rates, treatment costs and the increasing morbidity in the young population. In the past three decades, reperfusion strategies to alleviate ischemic injury have been widely included in clinical practice. However, reperfusion can lead to cardiomyocyte death, and subsequent irreversible myocardial damage.^1^

Myocardial ischemia-reperfusion injury (MIRI) is a long-term and complex pathophysiological process. The process of restoring blood flow to the ischemic myocardium can induce a wide range of basic biological changes, including impaired ion homeostasis, oxygen radical bursts, autophagy, ATP metabolic disorders, inflammation, oxidative stress, mitochondrial dysfunction, and apoptosis.^2^ Recent studies have demonstrated that the damage inflicted on the myocardium involves two processes: ischemia and reperfusion injury (I/R injury). I/R injury is a major determinant of long-term mortality; thus, the possibility of ameliorating the extent of the injury is of great individual and socioeconomic value.^3 - 7^

It has been demonstrated that cardiomyocyte apoptosis is a major mechanism of myocardial I/R-injury.^8^ Cardiomyocyte apoptosis is triggered by the mitochondrial and death receptor pathways under the conditions of myocardial reperfusion.^9^ However, these mechanisms have yet to be fully determined.^2^ Recent studies have explored the association between the endoplasmic reticulum (ER) and myocardial I/R-injury.^9 - 11^ Stress conditions such as I/R, hypoxia, and oxidative stress have been identified in the dysregulation of ER functions, thus triggering ER stress.^12^ The pathological consequences of ER lumen disruption and miscommunication have been implicated in myocardial I/R-injury.^13 , 14^ However, it is still unclear whether or not ER stress induces apoptosis by activating PERK during myocardial reperfusion injury.^15^

At present, due to the shortage of specific drugs and standard therapies, the timely and targeted treatment of I/R injury is often lacking. Dexmedetomidine (DEX) is a highly selective α_2_adrenergic receptor (AR) agonist, which has limited impact on hemodynamics and breathing, thus providing the ideal sedation and analgesia for patients undergoing cardiovascular surgery.^16^ Previous studies have demonstrated that DEX preconditioning showed cardioprotective effects in ischemic hearts.^17 - 20^ However, the molecular mechanism of DEX protection, to date, remains unknown. The present study hypothesized that pre-treatment with DEX protects the heart from I/R injury by PERK pathway activation, which is dependent on the activation of the α_2_receptor. Therefore, the present study used an isolated heart I/R model to evaluate the effects of DEX on I/R injury and its potential mechanism.

## Methods

### Experimental animals

Male adult Sprague-Dawley rats (8-10 weeks old; 260-280g) were obtained from the animal experiment center of Changsha (Changsha, China). All experimental procedures were in accordance with the “Guide for the Care and Use of Laboratory Animals”, published in China (No.1492, 2001), and were approved by the Experimental Animal Care and Use committee of the Zunyi Medical University. All rats were housed under standard conditions (room temperature, 22℃; 12 h light/dark cycle) with free access to food and water in the Guizhou Key Laboratory of Anesthesiology and Organ Protection of Zunyi Medical University.

### Isolation of adult rat hearts

Rats were anesthetized with sodium pentobarbital (45 mg/kg, intraperitoneally). After the successful anesthesia, the heart was immediately excised from the chest by sternotomy and immersed in ice-cold Krebs-Henseleit (K-H) solution (NaCl, 119 mM; KCl, 6.0 mM; CaCl_2_, 1.24 mM; NaHCO_3_, 20.1 mM; KH_2_PO_4_, 1.24 mM; MgSO_4_, 1.24 mM; glucose, 11.2 mM). The excised heart was retrogradely placed in a Langendorff perfusion setup via the aorta (PanLab). The K-H solution was perfused at a constant perfusion pressure of 75-80 mmHg and balanced with a mixture of 95% O_2_and 5% CO_2_to a pH of 7.35-7.45 at 37°C. A latex balloon connected to a pressure transducer was inserted in the left ventricle through the mitral valve, and filled with normal saline to produce a left ventricular end-diastolic pressure (LVEDP) of 2-10 mmHg. The balloon volume was maintained constant throughout the experiment. Heart rate (HR), LVEDP, left ventricular developed pressure (LVDP = LVSP - LVEDP), left ventricular pressure peak rates of positive and negative changes (±dp/dt_max_) and rate pressure product (RPP = HR x LVDP) were recorded by the Lab Chart system.

### I/R and DEX preconditioning in isolated rat heart

For each test, following a 15-minute equilibration of perfusion, the hearts in which the baseline LVDP and HR were >50mmHg and >200 beats/min were randomly assigned to five groups in a sealed envelope randomization assignment. The specimens in all groups were perfused for 30 minutes prior to 40 minutes of normothermic global ischemia, followed by 120 minutes of reperfusion with the exception of the control group (n= 6): (i) control group, no preconditioning protocol, perfused with normal K-H solution; (ii) I/R group, no preconditioning protocol, perfused with normal K-H solution prior to ischemia; (iii) DEX group, preconditioned with 10 nM DEX prior to ischemia; (iv) yohimbine (YOH) group, preconditioned with1 μM YOH prior to ischemia; and(v) DEX + YOH group, preconditioned with 10 nM DEX + 1μM YOH prior to ischemia. The concentrations of DEX (Hengrui pharmaceutical) and antagonist were selected based on previous studies. Yohimbine (YOH) was used as an α_2_-AR antagonist. DEX and YOH were dissolved in the modified Krebs-Ringer solution ( Figure 1 ).

**Figure 1:** – *Langendorff myocardial ischemia-reperfusion injury model. For each test, after a 15-minute equilibration of perfusion, the prepped hearts were randomly assigned to five groups for baseline LVDP and HR, which were less than 50 mmHg and 200 beats/min. The specimens in control group were continuously perfused with K-H solution for 205 minutes. The specimens in four other groups were perfused for 30 minutes prior to 40 minutes of normothermic global ischemia, followed by 120 minutes of reperfusion. The specimens in the I/R group had no preconditioning protocol perfused with normal K-H solution before ischemia; the specimens in DEX group were perfused with DEX (10 nM) before ischemia; the specimens in YOH group were perfused with YOH (1 μM) before ischemia; the specimens in DEX + YOH group were perfused with DEX (10 nM)+YOH (1 μM) before ischemia. LVDP: left ventricular developed pressure, HR: heart rate, K-H: Krebs-Henseleit, I/R: ischemia-reperfusion, DEX: dexmedetomidine, YOH: yohimbine*

### Infarct size determination

Infarct size was assessed by the triphenyl tetrazolium chloride (TTC) staining method. After reperfusion, the tissues that were adherent to the arterial root were removed, and the whole heart was frozen. The frozen heart was cut into 1-2 mm thick slices. The slices were immersed in a vial with 1% TTC and incubated in a water bath for 30 minutes at 37°C. The vial was continuously agitated to obtain an even staining. The slices were subsequently placed in 10% formaldehyde for 24 hours, placed on a glass plate, and a cover glass was placed over the slices. Shims of 2 mm were placed in the corners between the glasses to obtain the desired slice thickness, and digital photographs were taken. Areas of infarction and normal areas were determined in a blinded manner by planimetry with the Image-Pro Plus 6.0 software (Media Cybernetics, Inc.), and infarct size was expressed as a percentage of the total area.

### Histopathological examination

Myocardial tissues harvested from the rats were cut into 1 cm sections, fixed in 4% paraformaldehyde and embedded in paraffin. To quantify the extent of myocardial damage, the biopsies were cut into 5 µm sections, which were stained with hematoxylin and eosin (H&E) for 90 minutes, at room temperature, and six fields of view (magnification, x200) were randomly selected for evaluation in each group.

### TUNEL assay

Terminal deoxynucleotidyl transferase dUTP nick end labeling (TUNEL) assays were performed to determine the extent of apoptosis in the myocardium using a commercially available kit (Baiao Cisco Biological Technology) according to the manufacturer’s instructions. Green nuclear labeling was defined as TUNEL-positive cells. To determine the extent of myocardial apoptosis, five fields (magnification, x 200) were randomly selected from two sections in each group, and the apoptosis index (AI) was calculated using Image-Pro Plus 6.0 (AI) as follows: AI = number of apoptotic cells / total number of cells counted.

### Western blotting

The heart was homogenized and lysed with a RIPA buffer (Thermo Fisher scientific, Inc.). The tissues were centrifuged at 125 x g for six minutes and the supernatant was collected. The concentration of the protein was determined by a BCA protein assay (Thermo Fisher scientific, Inc.). The samples were separated by 10% sodium dodecyl sulphate polyacrylamide gel electrophoresis and transferred onto nitrocellulose membranes. The membranes were blocked with 5% skimmed milk for two hours, membranes were probed with anti-GRP78 (1:1000; cat no. PA1-014A; Thermo Fisher scientific), anti-PERK (1:1000; cat no. PA5-79193; Thermo Fisher scientific), anti-p-PERK (1:1000; cat no. MA5-15033; Thermo Fisher scientific), anti-eIF2α (1:1000; cat no. MA1-079; Thermo Fisher scientific), anti-p-eIF2α (1:1000; cat no. 44-728G; Thermo Fisher scientific), anti-ATF4 (1:1000; cat no. PA5-68802; Thermo Fisher scientific), anti-CHOP (1:1000; cat no. PA5-86145; Thermo Fisher scientific), anti-GAPDH (1:1000; cat no. MA5-32539; Thermo Fisher scientific) primary antibodies at 4°C overnight. After washing the membranes three times, the blots were incubated with horseradish peroxidase-conjugated secondary antibodies (1:1000; cat no. G-21234; Thermo Fisher scientific) for two hours. The content of the protein was determined by a gel imaging system (Tanon Science & Technology).

### Statistical analysis

The sample size of this study was based on the preliminary experiment. Means of LVEDP in the control group, the DEX group and the I/R group following reperfusion for 120 minutes were 12.52, 16.23, 22.39, respectively, and Standard deviations were 1.45, 2.44 and 145, respectively. The α-level test was considered as 0.05, Z0.05/2 = 1.96. The power level, 1 − β, was considered as 0.8. For the control and the EDX groups, a sample size of six was required for each group. For the I/R and the DEX groups, a sample size of four was required for each group. So, a sample size of six was determined per group.

Data are presented as mean ± standard deviation (SD). SPSS 20.0 (IBM, Corp) was used for statistical analyses. Differences between groups were analyzed with the one-way analysis of variance (ANOVA). The Shapiro-Wilk test was employed to verify normality. The LSD test was used for the comparison of homogeneous variance, and the Dunnett’s T3 test was used for the comparison of uneven variance. *P<* 0.05 was considered to indicate a statistically significant difference.

## Results

### DEX improves cardiac recovery from the I/R injury

To determine the effects of DEX on myocardial I/R-injury, an isolated perfused heart preparation was performed by perfusing rat hearts for 30 minutes with modified K-H solution prior to 40 minutes of global ischemia, followed by 120 minutes of reperfusion. The analysis of variance demonstrated there were no significant differences in hemodynamics between the groups at the end of the balance point. After 45 minutes of perfusion, no significant differences were observed in HR, ±dp/dt_max_, LVDP, LVEDP and RPP, with the exception of the YOH group and the DEX + YOH group. In the control group, HR, ±dp/dt_max_, LVDP, and RPP were higher and LVEDP was lower compared with the other groups after reperfusion for 120 minutes, which suggested that 40-minute ischemia at room temperature was challenging for rat hearts. During the second half of the 120-minute reperfusion, a number of hearts exhibited failure and arrhythmia (~10%). However, in the DEX group, HR, ±dp/dt_max_, LVDP, and RPP were higher and LVEDP was lower compared with the other groups after reperfusion for 120 minutes, with the exception of the control group. Therefore, DEX preconditioning significantly improved the hemodynamics following myocardial I/R injury, and the effects were reversed by the AR antagonist YOH ( Figure 2 ).

**Figure 2:** – *Dexmedetomidine improves cardiac function of ischemia-reperfusion rat hearts. A-F: effect of dexmedetomidine on the HR, rate pressure product (RPP = HR×LVDP), left ventricular pressure peak rates of positive and negative changes (±dp/dt_max_) of I/R injury in rats, LVDP, LVEDP. Data are presented as the mean ± standard deviation. n=12.^•^P<0.05, vs. ischemia beginning point,^#^P<0.05, vs. control group at reperfusion for 120 minutes.^∆^P<0.05, vs. I/R group at reperfusion for 120 minutes.^^P<0.05, vs. DEX group at reperfusion for 120 minutes. Baseline: at the end of the balance point, 45 minutes: ischemia beginning point, 205 minutes: 120 minutes of reperfusion. HR, heart rate, I/R: ischemia-reperfusion, LVDP: left ventricular developed pressure, RPP: rate pressure product, ±dp/dt_max_: left ventricular pressure peak rates of positive and negative changes, LVDP: left ventricular developed pressure, LVEDP: left ventricular end diastolic pressure, DEX: dexmedetomidine.*

### Pre-treatment with DEX reduces myocardial infarct size

TTC staining, which is the gold standard for testing myocardial infarct, was used to evaluate the cardio-protective function of DEX. In the control group, the percentage of infarct was low; however, it was significantly increased in the I/R, YOH and DEX + YOH groups ( Figure 3A ). In rats that were pre-treated with DEX, the infarct area was significantly smaller when compared with that in the I/R group. Additionally, no differences were observed between the YOH and the DEX + YOH groups compared with the I/R group. Therefore, the addition of YOH reversed the DEX-mediated protective effects on myocardial injury ( Figure 3 ).

**Figure 3:** – TTC staining and HE staining of Langendorff hearts after ischemia-reperfusion injury. (A) Representative images of TTC stained samples showing the infarct area (white) and the non-infarct area (red). (B) Analysis of the myocardial infarct size in control and in I/R-induced isolated heart. Data are presented as the mean ± standard deviation. n=6.^*^P<0.05, vs. control group;^#^P<0.05, vs. I/R group;^∆^P<0.05, vs. DEX group. (C) Representative images of HE stained samples (magnification, ×200) demonstrating histopathological changes in the myocardium. TTC: triphenyl tetrazolium chloride staining technique. HE: hematoxylin and eosin, I/R: ischemia-reperfusion.

### Pretreatment with DEX attenuates myocardial tissue damage

Myocardial injury was further measured in the H&E-stained sections. H&E staining revealed that myocardial structure was complete and exhibited regular arrangement, normal cardiac muscle fibers and no necrosis, with mild cardiomyocyte edema in the control group, whereas the structure of the myocardium was severely damaged after the I/R injury. The I/R group exhibited disordered myofibril arrangement and ruptured cardiac muscle fibers. As shown in Figure 3C , DEX preconditioning significantly improved these pathological changes; however, YOH partially reversed the protective effect.

### Pretreatment with DEX suppresses myocardial apoptosis

The TUNEL assay was used to determine the effects of DEX on cardiomyocytic apoptosis in isolated rat hearts after I/R. Compared with the control group (apoptotic rate, 0.00±0.00%), the number of TUNEL-positive cells increased significantly in the I/R (apoptotic rate, 58.17±0.60%), YOH (apoptotic rate, 57.11±1.39%) and DEX + YOH (apoptotic rate, 57.62±1.50%) groups, whereas no significant differences were observed in apoptotic rate among the I/R, YOH and the DEX + YOH groups ( Figure 4C ). Compared with the I/R, YOH and DEX + YOH groups, apoptotic rate was significantly decreased in the DEX group. Therefore, DEX may decrease myocardial apoptosis. However, YOH completely reversed this effect ( Figure 4 ).

**Figure 4:** – TUNEL assays to detect apoptosis in ischemia-reperfusion hearts. (A) Representative images of TUNEL assays (magnification, ×400) showing the total nucleus (blue) and apoptotic nucleus (green). (B) Analysis of the cardiomyocytic apoptotic nucleus in the control group and in I/R-induced isolated heart. (C) Analysis of the cardiomyocytic apoptosis rates in control group and in I/R-induced isolated heart. Data are presented as the mean ± standard deviation. n=6.^*^P<0.05, vs. control group;^#^P<0.05, vs. I/R group;^∆^P<0.05, vs. DEX group. TUNEL: Terminal deoxynucleotidyl transferase dUTP nick end labeling.

### DEX pret-reatment alleviates apoptosis by inhibiting the ER stress-mediated PERK pathway

To elucidate the successful establishment of an ER stress model induced by myocardial I/R injury, the activation of GRP78 and PERK was evaluated. In the present study, GRP78, which is an important marker for the occurrence of ER stress, was extensively upregulated at the protein level in all experimental groups compared with the control group ( Figure 5 ). As GRP78 is a direct target of PERK, which is a highly conserved ER stress-mediated apoptosis transducer, the expression levels of PERK and p-PERK protein were examined. Under physiological conditions, the overexpression of GRP78 did not change the activation of PERK as indirectly determined by PERK phosphorylation ( Figure 6 ). The expression of p-PERK protein in the I/R, YOH and DEX + YOH groups was higher compared with that in the control and the DEX groups ( Figure 6 ). By contrast, this was successfully blocked by the DEX treatment. These results suggested that DEX may participate in ER stress-mediated protection against reperfusion injury, whereas the protective effect of DEX was reversed by YOH compared with the DEX + YOH group. To further determine the effects of DEX on the PERK pathway and its potential molecular mechanism, downstream components of the PERK pathway in ER stress response were investigated, including the eukaryotic initiation factor 2α (eIF2α), activating transcription factor 4 (ATF4) and CCAAT/enhancer-binding protein homologous protein (CHOP). The results demonstrated that the levels of p-eIF2α, ATF4 and CHOP were notably upregulated in the I/R, YOH and DEX + YOH groups in comparison with the control group, whereas pre-treatment with DEX inhibited the expression of the three proteins ( Figure 5 , Figure 6 ). The examination by TUNEL assay confirmed these results. Therefore, the results indicated that DEX may relieve myocardial I/R injury-mediated apoptosis by suppressing the activation of the PERK pathway.

**Figure 5:** – Dexmedetomidine protected the myocardium from I/R injury and reduced apoptosis. (A) Western-blotting detecting of GRP78, ATF4, CHOP protein expression. (B-D) Analysis of the expression of ATF4, GRP78 CHOP in control and I/R-induced isolated heart. Data are presented as the mean ± standard deviation. n=6.^*^P<0.05, vs. control group.^#^P<0.05, vs. I/R group,^∆^P<0.05, vs. DEX group. I/R: ischemia-reperfusion. GRP78: glucose-related protein 78, ATF4: activating transcription factor 4, CHOP: CCAAT/enhancer-binding protein homologous protein.

**Figure 6:** – Effect of DEX on the expression of p-PERK and p-eIF2α. (A, B) Western-blotting detecting of PERK, p-PERK, eIF2α, p-eIF2α protein expression. (C, D) Analysis of the expression of PERK, p-PERK, eIF2α, p-eIF2α in control and I/R-induced isolated heart. Data are presented as the mean ± standard deviation. n=6.^*^P<0.05, vs. control group.^#^P<0.05, vs. I/R group,^∆^P<0.05, vs. DEX group. I/R: ischemia-reperfusion. PERK: protein kinase R-like ER kinase, p-PERK: phosphorylation-protein kinase R-like ER kinase, eIF2a: eukaryotic initiation factor 2a, p-eIF2α: phosphorylation-eukaryotic initiation factor 2a.

## Discussion

The present study indicated that DEX may protect against myocardial I/R injury, which was in accordance with a previous study.^21^ The protective effects of DEX on myocardial I/R injury through the inhibition of apoptotic pathways have been previously confirmed.^22^ Similarly, the results of the present study indicated that DEX improved cardiac hemodynamics and decreased apoptosis, which was revealed by the changes in apoptotic morphology and apoptotic rates in the TUNEL assay. Excessive ER stress has been demonstrated to serve an important role in myocardial I/R injury and lead to apoptosis.^23^ GRP78, which is an ER chaperone protein, belongs to one of the heat-shock protein 70 (Hsp70) family, which indicates the occurrence of ER stress and regulates ER homeostasis at a certain point.^24^ Previous studies have demonstrated that I/R induces the expression of GRP78 protein.^25 , 26^ The results of the present study demonstrated that the GRP78 protein was induced by I/R, whereas the DEX treatment decreased GRP78 expression, which indicated that DEX may exert its protective effects by suppressing ER stress.

PERK is a stress receptor on the ER with serine-threonine kinase activity, which is activated by trans-autophosphorylation and oligomerization. Under prolonged ER stress conditions, the PERK/eIF2α/ATF4/CHOP pathway contributes with apoptosis during myocardial I/R injury.^27 , 28^ A previous study has reported that membrane-localized GRP78 is crucial for PERK phosphorylation,^26^ which was also observed in the present study. It is reported that the highest expression of GRP78 protein was detected in the myocardium from the isolated and perfused rat heart with 40-minute ischemia and 120-minute reperfusion.^29^ In addition, we previously found that 40-minute ischemia was optimal for the Langendorff ischemia-reperfusion rat hearts,^30^ therefore, we selected 40-minute ischemia and 120-minute reperfusion in the present *ex vivo* ERS ischemia reperfusion injury model. It is also reported that the activation of the PERK kinase domain in the early stages of ER stress leads to the phosphorylation of eIF2α, which reduces transcriptional initiation and protein folding, and maintains homeostasis in the endoplasmic reticulum. Advanced phosphorylation of eIF2α through the activation of ATF4 induces the overexpression of the apoptosis protein CHOP, which leads to apoptosis.^31 , 32^ Related studies have shown that the mechanisms of DEX against myocardial I/R injury are mediated by the activation of α_2_-ARs and anti-inflammatory processes,^22^ whereas the effect of DEX on ER stress in myocardial I/R injury has rarely been investigated. The results of the present study have demonstrated that DEX inhibited the ER stress-mediated PERK/eIF2α/ATF4/CHOP pathway during myocardial I/R injury. A previous study has described that myocardial I/R causes abnormal accumulation of unfolded proteins in the lumen of the endoplasmic reticulum, and contributes with the autophosphorylation of PERK.^33^ In the present study, p-PERK protein expression level was increased in the I/R group, but decreased with the DEX pre-treatment. With the activation of PERK, the phosphorylation of downstream protein eIF2α inhibited 80S ribosome assembly and massive protein synthesis.^34 , 35^ Another study has indicated that the dephosphorylation of eIF2α suppressed downstream ATF4/CHOP signaling.^36^ Therefore, eIF2α may be a key component in PERK-mediated signaling. The results of the present study demonstrated that after the DEX treatment, the level of p-eIF2α was reduced compared with the I/R group. ATF4 plays a role in promoting apoptosis, which is induced by the phosphorylation of eIF2α.^37 - 39^ The results of the present study suggested that ATF4 may be responsible for cardiomyocyte apoptosis during I/R induced ER stress, whereas DEX treatment may downregulate ATF4. CHOP, which is a member of the CCAAT/enhancer binding protein family of transcription factors,^40^ is a classic marker of apoptosis initiation, which plays an important role in ER stress-mediated myocardial apoptosis.^41 , 42^ Our results suggested that the expression of CHOP protein increased in the I/R group, which corresponded to the results of the apoptosis assay. The present study demonstrated that DEX had an anti-apoptotic effect, and the mechanism may be dependent on the ER stress-mediated PERK pathway.

YOH is a selective blocker of presynaptic α_2_-AR, which dilates vascular smooth muscle, decreases sympathetic tone, and increases peripheral parasympathetic tone. In the present study, YOH reversed the protective effects of DEX pretreatment in the myocardium of rats, which resulted in increased myocardial infarct size and cardiomyocyte apoptosis; in addition, the expression levels of the PERK pathway proteins were increased. Together, these results indicated that the protective effect of DEX preconditioning in rat myocardium may be antagonized by an AR blocker, which is consistent with a previous study.^43^

Although the results of the present study identified an important role for the PERK pathway in the survival of cardiomyocytes, there were certain limitations to the study. Future work is necessary to analyze other potential mechanisms besides apoptosis and the PERK pathway. In addition, apoptosis is considered to occur in the context of excessive stress in the ER; by contrast, ER promotes normal cell survival by providing nutrients from digesting damaged organelles and proteins when ER stress levels are mild, which is termed ER stress-induced autophagy. Further analysis is required to determine the threshold between PERK exerting protective self-adaptation and injurious apoptosis in myocardial I/R injury.

## Conclusion

This study confirmed that DEX preconditioning reduced myocardial I/R injury and improved cardiac function by inhibiting PERK-mediated apoptosis pathway.
